# HIV-1 Conserved Elements p24CE DNA Vaccine Induces Humoral Immune Responses with Broad Epitope Recognition in Macaques

**DOI:** 10.1371/journal.pone.0111085

**Published:** 2014-10-22

**Authors:** Viraj Kulkarni, Antonio Valentin, Margherita Rosati, Morgane Rolland, James I. Mullins, George N. Pavlakis, Barbara K. Felber

**Affiliations:** 1 Human Retrovirus Pathogenesis Section, Vaccine Branch, Center for Cancer Research, National Cancer Institute at Frederick, Frederick, Maryland, United States of America; 2 Human Retrovirus Section, Vaccine Branch, Center for Cancer Research, National Cancer Institute at Frederick, Frederick, Maryland, United States of America; 3 Departments of Microbiology, Medicine and Laboratory Medicine, University of Washington, Seattle, Washington, United States of America; Tulane University, United States of America

## Abstract

To target immune responses towards invariable regions of the virus, we engineered DNA-based immunogens encoding conserved elements (CE) of HIV-1 p24^gag^. This conserved element vaccine is designed to avoid decoy epitopes by focusing responses to critical viral elements. We previously reported that vaccination of macaques with p24CE DNA induced robust cellular immune responses to CE that were not elicited upon wild type p55^gag^ DNA vaccination. p24CE DNA priming followed by p55^gag^ DNA boost provided a novel strategy to increase the magnitude and breadth of the cellular immune responses to HIV-1 Gag, including the induction of strong, multifunctional T-cell responses targeting epitopes within CE. Here, we examined the humoral responses induced upon p24CE DNA or p55^gag^ DNA vaccination in macaques and found that although both vaccines induced robust p24^gag^ binding antibody responses, the responses induced by p24CE DNA showed a unique broad range of linear epitope recognition. In contrast, antibodies elicited by p55^gag^ DNA vaccine failed to recognize p24CE protein and did not recognize linear epitopes spanning the CE. Interestingly, boosting of p24CE DNA primed animals with p55^gag^ DNA resulted in augmentation of antibodies able to recognize p24^gag^ as well as the p24CE proteins, thereby inducing broadest immunity. Our results indicate that an effectively directed vaccine strategy that includes priming with the conserved element vaccine followed by boost with the complete immunogen induces broad cellular and humoral immunity focused on the conserved regions of the virus. This novel and effective strategy to broaden responses could be applied against other antigens of highly diverse pathogens.

## Introduction

The development of an effective HIV vaccine is critical to control the AIDS pandemic. Protection against HIV is difficult to obtain in part because the virus readily mutates and generates viable alternatives to the epitopes targeted by the immune system of the host. To address the problem of viral variability many strategies have been tested [1]–[19]. We have focused in conserved elements from the HIV-1 proteome and have based our immunogen designs on (a) stringent conservation among all HIV sequences, (b) selection of epitopes associated with better virologic control in HIV-1 infected individuals, and (c) optimizing immunogen expression and proteolytic cleavage [11], [12], [16]–[25]. We focused on Gag as a prototype vaccine, because Gag-specific CD8^+^ T cell responses were found to correlate with control of viremia in clade B and C infected individuals [26]–[29], to contribute to control HIV-1 after infection in the Step trial [30], and Gag-specific CD4^+^ T cell responses were associated with virus control [31], [32]. We selected 7 conserved elements (CE) within HIV-1 p24^gag.^ which cover ∼98% of the HIV-1 Group M viruses worldwide and represent 54% of the p24^gag^ protein. These p24CE sequences were collinearly arranged to express what we refer to as the p24CE immunogen [18]. Using *ex vivo* transfection of CE-encoding RNA into human dendritic cells, we were able to generate levels of CD4 and CD8 responses comparable to that of the full-length p55^Gag^ protein [25]. C57BL/6 mice immunized with p24CE plasmids developed immune responses of high magnitude and recognized more CE than those induced by the complete p55^gag^ DNA immunogen [18]. Vaccination of macaques with p24CE DNA vaccine showed that all of the 10 animals developed cellular responses against the CE, while only 50% of the macaques vaccinated with the full-length p55^gag^ DNA developed cellular responses targeting any of the CE [19]. Importantly, boosting CE-primed macaques with DNA expressing full-length p55^gag^ increased magnitude and poly-functionality of the CE responses, and breadth of Gag immunity, targeting epitopes within the highly conserved elements and also outside p24^gag^, and demonstrating alteration of the hierarchy of epitope recognition in the presence of pre-existing CE-specific responses [19].

Although the p24CE vectors were primarily designed as a prototype T cell vaccine to induce optimal cell-mediated responses, the development of humoral immunity is critical to contain HIV transmission. Therefore, we explored whether the CE vaccine approach also elicits humoral immunity and defined the breath of the antibody responses observed. Although antibodies targeting the HIV Gag protein are not expected to prevent viral transmission, this study serves as proof-of-concept that can be translated to other viral antigens, such as Env. In the present work, we performed a detailed analysis of the antibodies induced by p24CE DNA vaccination in macaques [19], including peptide mapping of the different CE that are recognized by those antibody responses. We report that the p24CE DNA vaccine was able to induce robust humoral immune responses that recognized wild type HIV-1 p24^gag^. Interestingly, we also found that the CE-induced responses were broad, sustained, and could be boosted by heterologous p55^gag^ DNA vaccination. Thus, our finding suggests that inclusion of conserved elements as a priming immunogen provides a novel and effective strategy to broaden responses against highly diverse pathogens like HIV by avoiding decoy epitopes, while focusing responses to critical viral elements for which fewer escape pathways exist. This study serves as proof-of-concept to assess immunogenicity of conserved elements in the macaque model, a concept that can be translated into other viral proteins, such as Env, where the combination of distinct antibody and cellular responses against invariable epitopes may provide an immunologic advantage for the containment of the virus.

## Material and Methods

### Ethics statement

The animals in this study were Indian rhesus macaques (Macaca mulatta) as described previously [19] and were housed at the Advanced BioScience Laboratories, Inc. (ABL) animal facility. All animals were cared for and procedures performed under a protocol approved by the ABL Animal Care and Use Committee (animal welfare assurance no. A3467-01; protocol no. AUP516) and USDA Certificate number 51-R-0059. Furthermore, the macaques in this study were managed according to the animal husbandry program of the ABL Animal Facility, which aims at providing consistent and excellent care to nonhuman primates at the vivarium. This program operates based on the laws, regulations, and guidelines promulgated by the United States Department of Agriculture (e.g., the Animal Welfare Act and its regulations, and the Animal Care Policy Manual), Institute for Laboratory Animal Research (e.g., Guide for the Care and Use of Laboratory Animals, 8th edition), Public Health Service, National Research Council, Centers for Disease Control, and the Association for Assessment and Accreditation of Laboratory Animal Care (AAALAC) International. The nutritional plan utilized by the ABL Animal Facility consisted of twice daily feeding of Labdiet 5045 High Protein Primate Diet and food intake was closely monitored by Animal Research Technicians. This diet was also supplemented with a variety of fruits, vegetables, and other edible objects as part of the environmental enrichment program established by the Veterinary staff and enrichment Technician. Pairing of animals as part of the environmental enrichment program was managed by the enrichment technician. All primary enclosures and animal rooms were cleaned daily with water and sanitized at least once every two weeks. All macaques (N = 10) used in this study were males, except for four females: M695, P572, P574, M437. Their average weight was 8.5 kg (range: 3.8–11.2 kg) and their average age was 7 years (range: 4–12 years). Vaccinations were performed under anesthesia (Ketamine administered at 10 mg/kg) and all efforts were made to minimize suffering. No adverse effects were found. None of the animals were euthanized as part of this study.

### Vaccination regimens

The macaques were vaccinated as part of a previous study [19] with plasmids expressing p24CE1 and p24CE2 or COT-M p55^gag^ DNA, and all the vaccines contained IL-12 DNA, described in detail elsewhere [18], [19]. The DNA vaccines were delivered via intramuscular injection followed by *in vivo* electroporation [19]. The animals received two vaccinations with p24CE DNA (N = 10) or p55^gag^ DNA (N = 4) (vaccination 1, vaccination 2) followed by a converse boost with p55^gag^ DNA or p24CE DNA (vaccination 3), respectively.

### Antibody assays

Blood samples were collected at the day of each vaccination and 2 weeks later. Serial dilutions of plasma samples were tested by standard ELISA (Advanced Bioscience Lab, Rockville, MD) for binding antibodies to HIV-1 clade B p24^gag^ and the absorbance (OD) at 450 nm was determined. Binding titers were reported as the reciprocal of the highest dilution scoring positive (having a value greater than average values obtained with naive macaque plasma +3 standard deviations). Pepscan analysis was performed using 58 peptides covering HIV-1 consensus group M p24^gag^ (15-mers overlapping by 11 AA; #11057 AIDS Research and Reference Reagent Program, Germantown, MD) by standard ELISA. The plasma samples were tested at a dilution of 1:50 and absorbance (OD) was measured at 450 nm.

For Western blot analysis, 10^6^ HEK293 cells were transfected with 1 µg of the p24CE DNAs or 5 µg of the HIV-1 molecular clone pNL4-3. Aliquots of the extracellular fractions containing p24^gag^ (pNL4-3; 1/250) or cell-associated fractions containing p24CE1 and p24CE2, respectively (1/50 of extract) were separated on 12% NuPAGE gels and blotted onto nitrocellulose membranes (Invitrogen, Carlsbad, CA). The membranes were incubated with plasma from vaccinated macaques, followed by anti-monkey–HRP labeled antibody (dilution 1:10,000), and the bands visualized using the enhanced chemiluminescence (ECL) Prime Western blotting detection reagent (GE HealthCare, Piscataway, NJ).

## Results

### Development of humoral immune responses in macaques vaccinated with p24CE and p55^gag^ plasmid DNA

**Figure 1:**
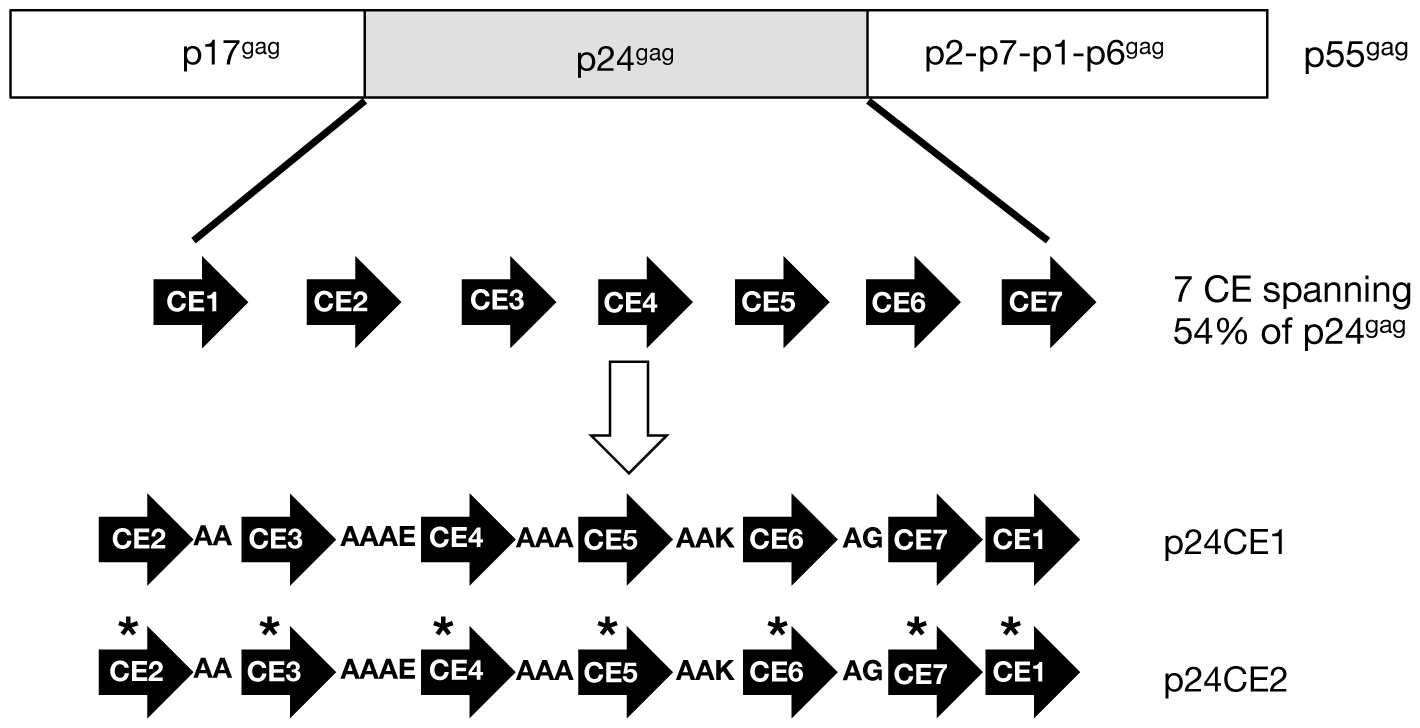
p24CE Expression plasmids. Cartoon depicting the HIV p55^gag^ protein and its proteolytic cleavage products p17^gag^, p24^gag^ and the C-terminal p2, p7, p1 and p6 proteins. The 7 highly conserved elements (CE) identified within p24^Gag^ are indicated. The two p24CE constructs differ by 1 ‘toggle’ AA per CE, indicated by asterisks. The 7 CE were connected via linkers and the p24CE proteins were expressed from the CMV promoter using the mammalian expression vector pCMV.kan [18].

To focus the immune responses on nearly invariant regions of the HIV-1 proteome, we developed DNA immunogens encoding 7 highly conserved elements (CE) identified within HIV-1 p24^gag^ [18], [19], [23]. Two plasmids were constructed with each of the 7 CE elements, which differed by 1 ‘toggle’ amino acid per CE to include a lesser conserved AA, and were arranged by taking into consideration the hydrophobicity of individual CE and spaced with linkers designed to maximize cleavage between the adjacent CE (**Figure 1**) as described previously [18], [19], [23]. Each 140-AA CE cassette, p24CE1 and p24CE2, was expressed from expression-optimized DNA vectors.

**Figure 2:**
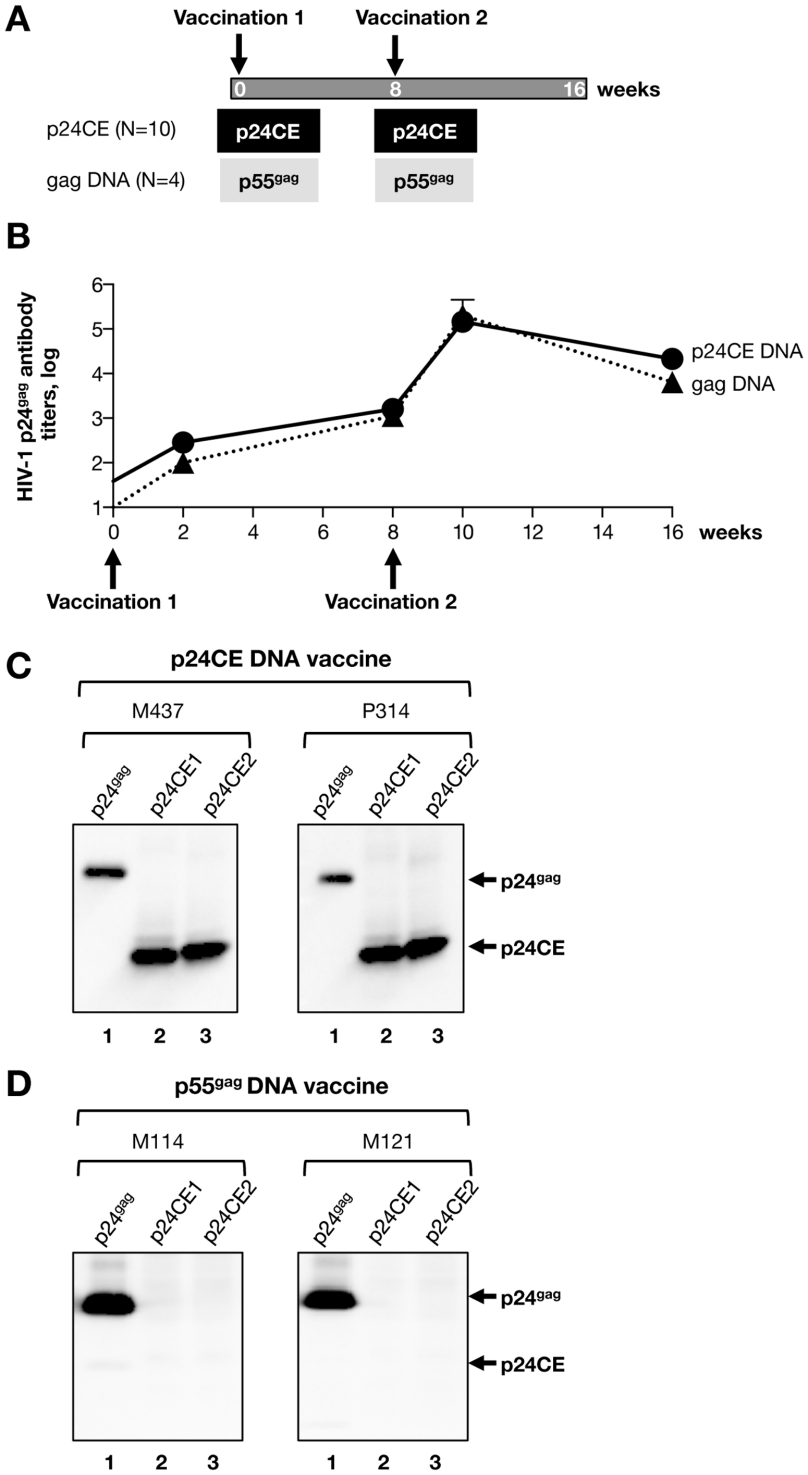
Induction of humoral immune responses upon p24CE DNA vaccination in macaques. **(A)** Cartoon depicts the vaccination schedule. Each macaque received 2 vaccinations with a mixture of p24CE1 and p24CE2 DNAs or p55^gag^ DNA at week 0 and 8. Plasma samples were analyzed for bAb to Gag at the time of each vaccination and at the additional indicated time points. **(B)** Plasma bAb to HIV-1 p24^Gag^ measured by ELISA. Mean and standard error of the reciprocal Gag antibody endpoint titers (in log_10_) of 10 macaques vaccinated with p24CE DNA mixture and 4 macaques which received the p55^gag^ DNA are shown. (**C, D**) Western immunoblot analysis was used to identify binding antibodies to HIV-1 p24^Gag^ and the p24CE proteins present in plasma collected after the last vaccination. The membranes contain the soluble processed clade B p24^gag^ protein (lane 1), p24CE1 (lane 2) and p24CE2 (lane 3) protein and were probed with plasma collected after the last vaccination. The data from 2 representative macaques from each vaccine group are shown: **(C)** p24CE DNA vaccinated animals M437 (plasma dilution 1:2000) and P314 (plasma dilution 1:1000) and **(D)** p55^gag^ DNA vaccinated animals M114 and M121 (plasma dilution 1:2000 for both). The positions of p24^gag^ and p24CE proteins are indicated.

Rhesus macaques received two vaccinations with DNAs expressing either both p24CE1 and p24CE2 (N = 10) or p55^gag^ (N = 4), each delivered by intramuscular injection followed by *in vivo* electroporation as outlined in **Figure 2A** and previously reported [18], [19]. Plasma samples were collected on the day of vaccination and 2 and 8 weeks later. We have previously reported that vaccination with p24CE DNA induced strong cellular immunity targeting new epitopes [18], [19]. Here, we examined the development of Gag-specific humoral responses induced by the same immunization regimen. Animals vaccinated with p24CE DNA developed antibody responses against p24^gag^ and the kinetics of these responses were similar to those induced by p55^gag^ DNA vaccination (**Figure 2B**). p24^gag^ antibody responses were detectable upon a single DNA vaccination, and were efficiently boosted by the 2^nd^ DNA immunization resulting in peak antibody titers (∼5.3 log reciprocal end-point dilution) detected 2 weeks later. Similar levels of Gag-specific antibody responses were detected 8 weeks later in both groups of animals, indicating comparable persistence of humoral responses. These results demonstrate that p24CE DNA, an immunogen designed to elicit optimal T-cell responses, is able to induce humoral immune responses to p24^gag^ of similar magnitude as those induced by the p55^gag^ DNA vaccine.

The ability to recognize the p24CE proteins and processed p24^gag^ by the antibodies induced by either of the vaccination regimens was analyzed by Western immunoblots (**Figure 2C–D**). Plasma from macaques vaccinated with p24CE DNA contained antibodies that strongly reacted with processed p24^gag^ and both forms of the p24CE proteins (p24CE1 and p24CE2) (**Figure 2C**). As expected from the conservation within p24CE of the AA sequences from the HIV-1 M group, these antibodies strongly reacted with Gag from different subtypes, including subtypes A, B, C, and group M consensus (not shown). In contrast, plasma samples from macaques immunized with p55^gag^ DNA, although able to recognize the viral p24^gag^, failed to bind to either of the two CE proteins (**Figure 2D**). Similar patterns of antibody recognition were found upon vaccination of mice [18]. Thus, antibodies induced by p24CE DNA vaccination recognized both the full-length p24^Gag^ and the p24CE proteins, but immunization with p55^gag^ DNA vaccination failed to produce antibodies specific for the CE proteins of p24^gag^.

### Humoral responses targeted four of the seven CE

**Figure 3:**
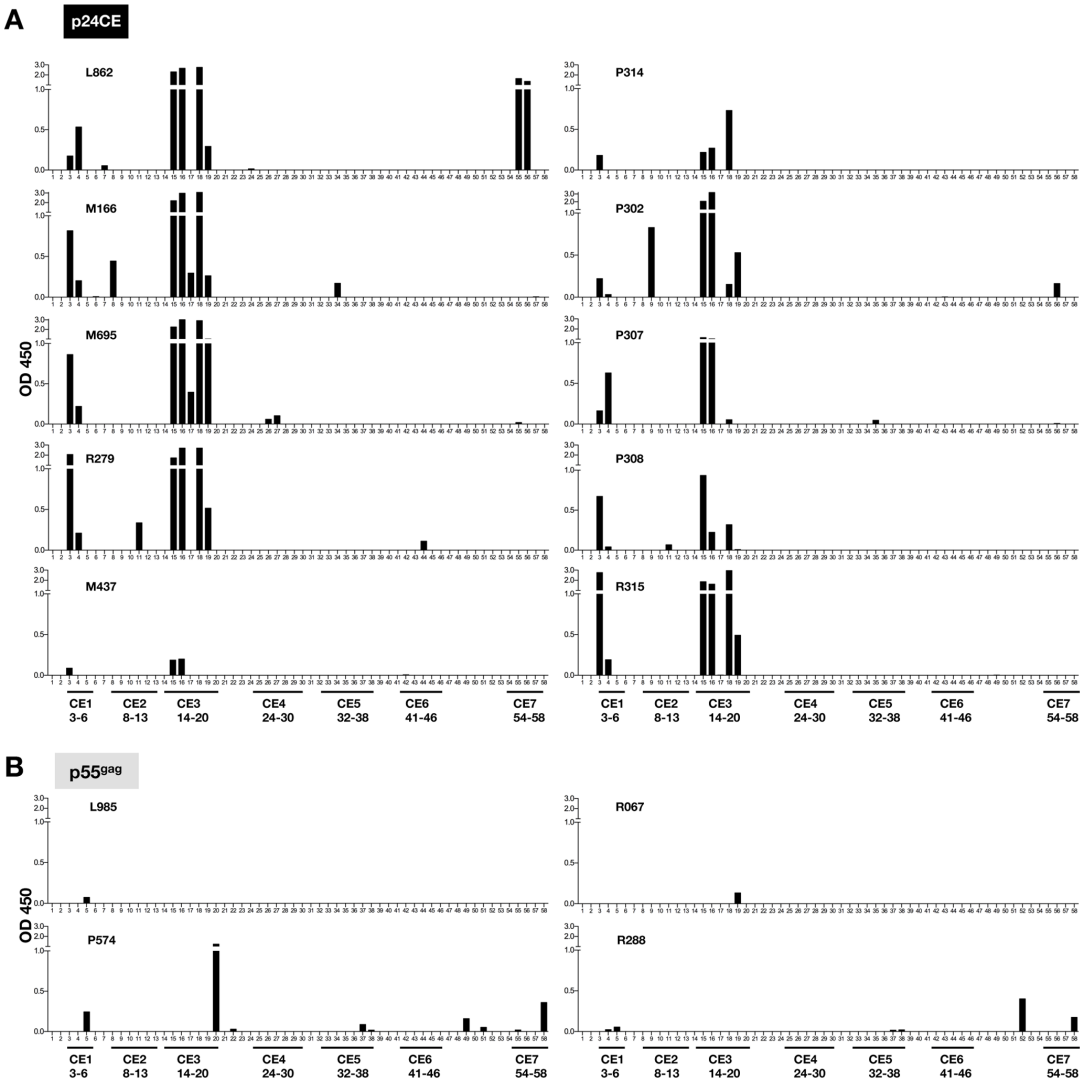
Pepscan analysis of humoral responses induced upon p24CE and p55^gag^ DNA vaccination in macaques. All macaques vaccinated with **(A)** p24CE DNA (N = 10) and **(B)** p55^gag^ DNA (N = 4) were subjected to Pepscan analysis against 58 peptides (15-mers overlapping by 11 AA) spanning p24^gag^. Peptides corresponding to CE1 to CE7 are indicated and the peptides are listed below as detailed in **Table 1**. OD_450_ is reported after subtracting the mean OD_450_ of the values obtained from negative samples plus 3 SD.

To identify the linear epitopes recognized by the antibodies induced by the vaccination regimens, we performed a peptide scanning analysis (Pepscan) of plasma samples at the time of peak antibody responses (2 weeks after the 2^nd^ vaccination) using a panel of peptides (15-mers overlapping by 11 AA) that cover the entire p24^gag^ protein (see **Table 1** for peptide sequences and association with CE). Data obtained with samples from macaques vaccinated with the p24CE DNA showed that each of the 10 animals developed antibodies that recognized CE1 and CE3 (**Table 2**). In addition, the number and locations of the peptides recognized suggest two distinct immunogenic epitopes within CE3. Three macaques (M166, R279 and P302) also developed responses to CE2, while two animals had responses to CE7 (L862, strong responses; P302, low responses) (**Figure 3A**). As expected, none of the macaques immunized with the p24CE recognized any peptide located outside the p24CE. In contrast, we found that the antibodies induced by p55^gag^ DNA vaccination failed to efficiently recognize linear epitopes within the p24^gag^ region (**Figure 3B**). Only one of the 4 animals (P574) had antibodies that recognized linear epitopes in CE1, CE3 and CE7, but we noted that different peptides were recognized compared to those in p24CE DNA vaccinated animals. Two macaques (P574 and R288) were able to recognize some peptides located in the ‘variable’ region between CE6 and CE7. These results demonstrate that vaccination with p24CE DNA triggered broad antibody responses that recognized several continuous epitopes within p24^Gag^, whereas vaccination with p55^gag^ DNA failed to elicit antibodies against the linear segments in the conserved regions or in HIV-1 p24^gag^ in general.

**Table 1:** Peptide list and coverage of CE.

ID	Peptide^a^	CE		ID	Peptide^a^	CE
1	PIVQNLQGQMVHQAI			31	WMTSNPPIPVGEIYK	
2	NLQGQMVHQA**ISPRT**			32	NPPIPVGEIY**KRWII**	CE5
3	GQMVHQA**ISPRTLNA**	CE1		33	PVGEIY**KRWIILGLN**	CE5
4	HQA**ISPRTLNAWVKV**	CE1		34	Y**KRWIILGLNKIVR**	CE5
5	**SPRTLNAWVKV**IEEK	CE1		35	**WIILGLNKIVRMYSP**	CE5
6	**LNAWVKV**IEEKAFSP	CE1		36	**GLNKIVRMYSPVSI**L	CE5
7	**VKV**IEEKAFSPE**VIP**	CE2		37	**IVRMYSPVS**ILDIRQ	CE5
8	EEKAFSPE**VIPMFSA**	CE2		38	**YSPVSI**LDIRQGPK	CE5
9	FSPE**VIPMFSALSEG**	CE2		39	**VSI**LDIRQGPKEPFR	
10	**VIPMFSALSEGATPQ**	CE2		40	DIRQGPKEPFRD**YV**	
11	**FSALSEGATPQDLN**T	CE2		41	RQGPKEPFRD**YVDRF**	CE6
12	**SEGATPQDLN**TMLNT	CE2		42	KEPFRD**YVDRFFKTL**	CE6
13	**TPQDLN**TMLNT**VGGH**	CE2		43	RD**YVDRFFKTLRAEQ**	CE6
14	LNTMLNT**VGGHQAAM**	CE3		44	**DRFFKTLRAEQA**TQ	CE6
15	LNT**VGGHQAAMQMLK**	CE3		45	**FKTLRAEQA**TQDVKN	CE6
16	**GGHQAAMQMLKDTIN**	CE3		46	**RAEQA**TQDVKNWMT	CE6
17	**AAMQMLKDTINEEAA**	CE3		47	**EQA**TQDVKNWMTDTL	
18	**MLKDTINEEAAEWDR**	CE3		48	TQDVKNWMTDTLLVQ	
19	**TINEEAAEWDR**LHPV	CE3		49	KNWMTDTLLVQNANP	
20	**EAAEWDR**LHPVHAGP	CE3		50	TDTLLVQNANPDCKT	
21	**WDR**LHPVHAGPIPPG			51	LVQNANPDCKTILKA	
22	HPVHAGPIPPGQMR			52	ANPDCKTILKALGPG	
23	HAGPIPPGQMRE**PRG**			53	CKTILKALGPGATL	
24	IPPGQMRE**PRGSDIA**	CE4		54	ILKALGPGAT**LEEMM**	CE7
25	GQMRE**PRGSDIAGTT**	CE4		55	LGPGAT**LEEMMTACQ**	CE7
26	EP**RGSDIAGTTSTLQ**	CE4		56	AT**LEEMMTACQGVGG**	CE7
27	**SDIAGTTSTLQEQIA**	CE4		57	**EMMTACQGVGGPGHK**	CE7
28	**GTTSTLQEQIAW**MTS	CE4		58	**ACQGVGGPGHK**ARVL	CE7
29	**TLQEQIAW**MTSNPPI	CE4				
30	**EQIAW**MTSNPPIPVG	CE4				

a Amino acids indicated in bold cover the respective CE.

**Table 2:** Responses of individual CE to cellular an humoral immune responses.

Vaccine	Macaques (N = 10)		Cellular^a^ and humoral immune responses to individual CE						
			CE1	CE2	CE3	CE4	CE5	CE6	CE7
Conserved Element p24CE DNA	L862	CMI^a^		**+**	**+**		**+**		
		pepscan	**+**		**+**				**+**
	M166	CMI^a^		**+**			**+**	**+**	
		pepscan	**+**	**+**	**+**		**+**		
	M695	CMI^a^			**+**		**+**	**+**	
		pepscan	**+**		**+**	**+**			
	R279	CMI^a^				**+**	**+**	**+**	
		pepscan	**+**	**+**	**+**				
	P314	CMI^a^			**+**	**+**	**+**		
		pepscan	**+**		**+**				
	M437	CMI^a^					**+**		
		pepscan	**+**		**+**				
	R315	CMI^a^					**+**		**+**
		pepscan	**+**		**+**				
	P302	CMI^a^			**+**		**+**	**+**	
		pepscan	**+**	**+**	**+**				**+**
	P307	CMI^a^			**+**		**+**	**+**	
		pepscan	**+**		**+**				
	P308	CMI^a^			**+**		**+**		
		pepscan	**+**		**+**				
Positive responses among 10 macaques		CMI^a^	0	2	6	2	10	5	1
		pepscan	10	3	10	1	1	0	2

a Cellular immune response data are from [19].

### Heterologous boost vaccination increased pre-existing antibody responses in p24CE and p55^gag^ DNA primed macaques

**Figure 4:**
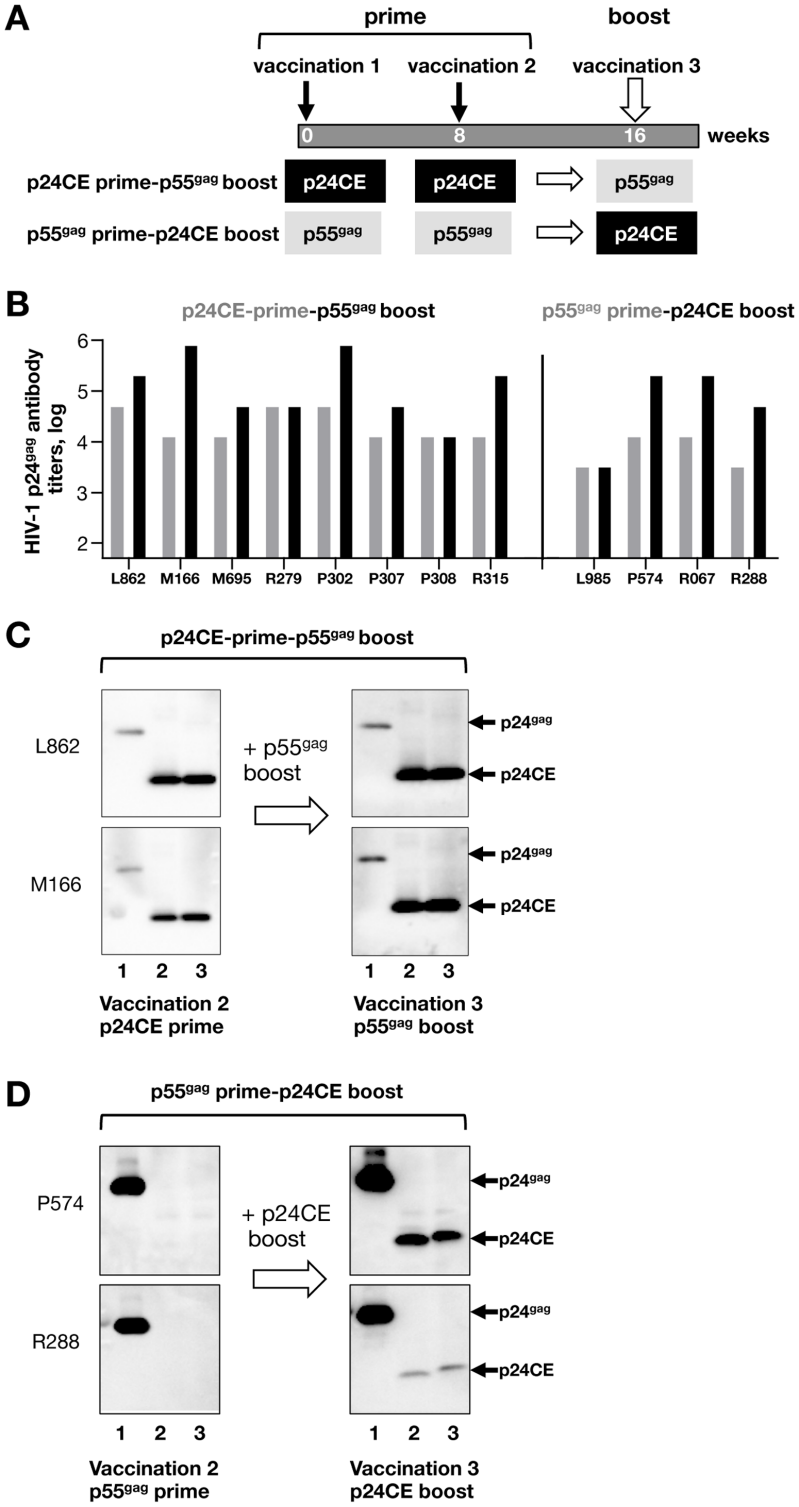
HIV-1 p55^gag^ DNA vaccination did not prime *de novo* CE humoral responses but increased pre-existing CE-specific responses. **(A)** Cartoon depicts the vaccination schedule. After receiving p24CE or p55^gag^ DNA as priming vaccination (see **Figure 2**) the animals received a heterologous boost vaccination (vaccination 3) with p55^gag^ DNA or p24CE DNA, respectively. **(B)** Comparison of plasma bAb to HIV-1 p24^gag^ ELISA titers at vaccination 3 and 2 weeks later. The reciprocal Gag antibody endpoint titers (in log_10_) from individual macaques vaccinated with the p24CE DNA mixture following by a p55^Gag^ DNA boost (N = 10; left panel) and macaques vaccinated with p55^Gag^ DNA followed by a p24CE DNA boost (N = 4; right panel) are shown. Data from day of 3^rd^ vaccination are from Fig. 2B. **(C, D)** Western immunoblot analysis was used to test macaque plasma for binding antibodies before (after vaccination 2) and after the boost (after vaccination 3). The membranes contain the soluble processed p24^gag^ (lane 1), p24CE1 (lane 2) or p24CE2 (lane 3) proteins and were probed with plasma collected 2 weeks after vaccinations 2 and 3. Data from 2 representative macaques are shown from each vaccine group. The membranes were probed with plasma from **(C)** macaques (L862, M166), which received the p24CE prime-p55^gag^ DNA boost (plasma dilution 1:2000) and from **(D)** macaques (P574, R288), which received the p55^gag^ prime-p24CE DNA boost regimen (plasma dilution 1:500).

We next asked whether boosting with the heterologous DNA plasmid could increase pre-existing humoral responses. Animals from both groups received an additional DNA vaccination as outlined in **Figure 4A** and the responses were monitored by ELISA. Sustained antibody responses were observed at the day of the heterologous boost vaccination in both groups (**Figure 4B**, vaccination 3) as a result of the prior priming vaccination. Plasma samples collected 2 weeks later showed that these responses were further boosted by heterologous DNA vaccination in both vaccine groups. Boosting of p24CE DNA primed responses by p55^gag^ DNA resulted in an average 0.8 log increase in ELISA titers, and boosting of p55^gag^ DNA primed responses by p24CE DNA increase the Gag ELIA titers by 0.9 log.

The responses were further analyzed by Western immunoblot (**Figure 4C, D**). Prior to boost, macaques primed with p24CE recognized the naturally processed p24^gag^ as well as the p24CE1 and p24CE2 proteins (**Figure 4C**). Following the heterologous p55^gag^ DNA boost, stronger reactivity was found against both p24^gag^ and p24CE proteins and the data from two representative animals (L862 and M166) are shown (**Figure 4C**). Interestingly, although p55^gag^ priming was unable to induce detectable antibody responses to the CE proteins by Western blot analysis, it was able to boost preexisting CE-specific humoral responses.

Macaques primed with p55^gag^ DNA (2 weeks after the 2^nd^ vaccination) developed humoral responses that strongly reacted with p24^gag^ but failed to recognize p24CE proteins (**Figure 4D**), as expected (see above **Figure 2D**). However, a single boost of these animals with p24CE DNA not only increased the reactivity to p24^gag^, but it also elicited antibodies recognizing p24CE proteins as shown by Western immunoblot analysis from two representative animals (P574 and R288) (**Figure 4D**).

**Figure 5:**
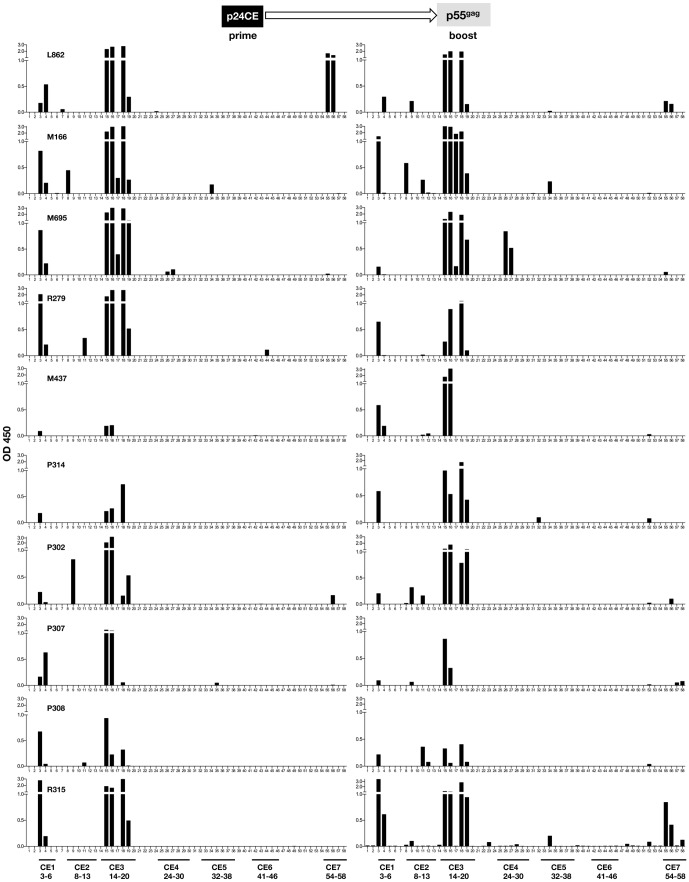
Pepscan analysis of humoral responses induced upon heterologous prime-boost regimens of macaques primed with p24CE priming boosted p55^gag^ DNA boost vaccination. Plasma collected after the prime and after the boost vaccinations were subjected to Pepscan analysis against the 58 overlapping peptides described in **Figure 3**. Peptides corresponding to CE1 to CE7 are indicated and the peptides are listed below as detailed in **Table 1**. Samples were analyzed after the priming (data from Figure 3A, left panels) and boost vaccination (right panels).

We also compared the antibody responses by Pepscan analysis of plasma samples collected after the prime (from **Figure 3**) and after the boost (**Figures 5** **and** **6**). In macaques primed with p24CE and boosted with p55^gag^ DNA (**Figure 5**), we observed no significant changes in the reactivity to linear epitopes except for M695, M437, P314 and R315, which showed increased recognition of 1 or 2 CE (M695:CE4; M437: CE1 and CE2; P314: CE1 and CE3) or new responses (R315: CE5 and CE7). Although p55^gag^ DNA vaccination did not induce antibodies against many linear peptides (see **Figure 3B**), it is noteworthy that it was able to alter responses in 4 of 10 animals, suggesting that it increased pre-existing low peptide recognition rather than inducing *de novo* responses.

**Figure 6:**
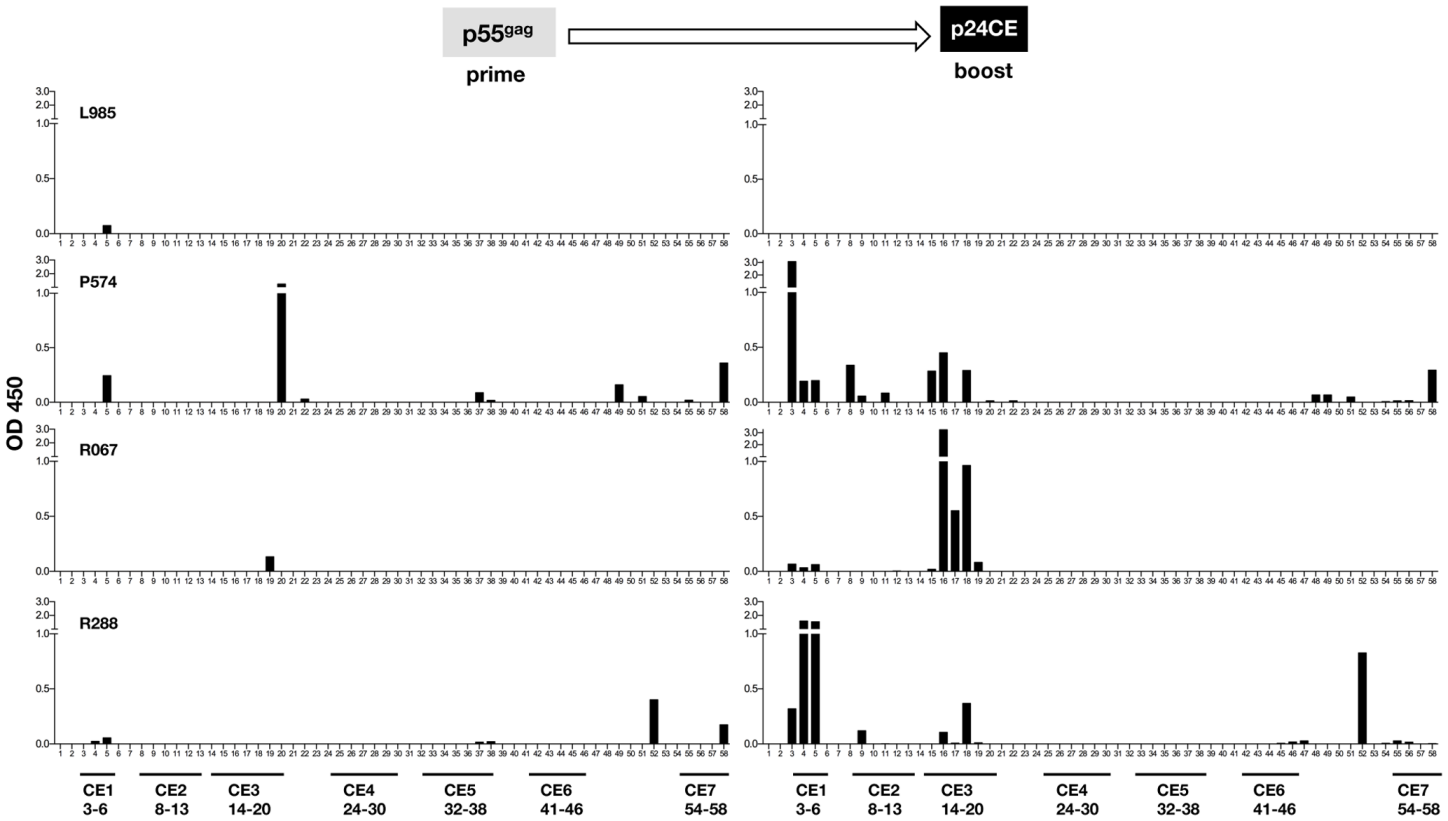
Pepscan analysis of humoral responses induced upon heterologous prime-boost regimens of macaques primed with p55^gag^ DNA and boosted with p24CE DNA. Plasma collected after the prime (left panels) and after the boost vaccinations (right panels) were subjected to Pepscan analysis as described in **Figures 3** **and** **5**.

In contrast, p55^gag^ primed macaques boosted with p24CE DNA (**Figure 6**) showed *de novo* production of antibodies recognizing multiple CE in 3 of 4 macaques, with the linear peptide recognition pattern found from p24CE primed animals (see **Figure 3A**). These macaques (P574, R067 and R288) developed antibody responses mainly targeting CE1 and CE3, the two CE most commonly recognized upon the p24CE vaccination. Macaque L985 was the only animal that did not develop antibodies against CE after a single p24CE DNA boost.

## Discussion

A successful vaccine against HIV-1 should induce both cellular and humoral immunity. We previously demonstrated that the p24CE vaccine induced strong cellular responses in both mice and macaques [18], [19]. These responses were mediated by both CD4^+^ and CD8^+^ T cells with cytotoxic potential and targeted several of the CE encoded by the vaccine. In this report, we tested whether an immunogen designed to induce efficient cellular immune responses against conserved regions of HIV-1 p24^gag^ was also able to induce antibody responses in vaccinated rhesus macaques. We found that vaccination of macaques with p24CE also induced anti-Gag humoral responses with binding antibody titers and kinetics similar to those induced by p55^gag^ DNA vaccination. Interestingly, we found that the antibodies elicited by the p24CE DNA vaccine are able to recognize the processed p24^gag^, whereas antibodies induced by p55^gag^ DNA vaccination failed to recognize the p24CE protein.

In addition, humoral responses induced by vaccination with the p24CE vectors targeted multiple linear epitopes within the CE, while antibodies induced by the p55^gag^ vaccine failed to recognize any of the conserved elements and in general only poorly induced antibodies able to recognize linear epitopes. Similar to the induction of cellular immunity [19], the p55^gag^ DNA vaccine was able to boost pre-existing CE-specific humoral responses in p24CE DNA primed animals. Table 2 shows a summary of the mapping of both cellular and humoral immune responses observed in the vaccinated animals. It is noteworthy that all the CEs selected for inclusion in the vaccine were immunogenic (humoral, cellular or both) in macaques, although the epitopes were selected based on association with better virologic control in HIV-1 infected individuals [23], [24]. CE5 (YVDRFYKTLRAEQA) induced cell- mediated responses in all the vaccinated animals, but was poor in inducing humoral responses. In contrast, CE1 (VIPMFSALSEGATPQDLN) elicited humoral responses in all the animals but failed to induce cellular responses in any of the vaccinated macaques. Interestingly, all macaques developed antibody responses against CE3 (PRGSDIAGTTSTLQEQIGW), while several vaccinees (6 of the 10 macaques) also induced strong cellular immunity against this region, suggesting that CE3 is a good immunogen for both arms of the immune system in macaques. Hence, CE3, CE5 and CE6 (LEEMMTACQGVGGPGHK) express the epitopes most efficiently recognized by the cellular immunity, while CE1 and CE3 are most efficient in inducing humoral responses.

In summary, these results demonstrate that an immunogen designed to induce cellular immune responses, and shown to do it efficiently in macaques [19], can also induce broad and distinct humoral immunity (this report). Although a correlation between humoral responses to Gag and improved virus control has been reported [33]–[35], future development of this vaccine concept will, logically, also include Env. It is further possible that analogous immunogens may benefit from both cellular immunity and the development of antibodies that otherwise may not be induced by the native molecule. Antibodies targeting linear epitopes could be useful in mediating protection either by binding to incoming viruses at mucosal surfaces or by mediating ADCC of infected cells. The combination of cellular and humoral responses targeting invariable regions of the virus may be beneficial to achieve protection against HIV-1 infection. Inclusion of conserved elements as a priming immunogen provides an effective vaccine to increase the breadth of the immune response. The concept of focusing responses to critical viral elements for which fewer escape pathways exist and of avoiding decoy epitopes could also be applied against other antigens of highly diverse pathogens.
